# Characteristic patterns of inter- and intra-hemispheric metabolic connectivity in patients with stable and progressive mild cognitive impairment and Alzheimer’s disease

**DOI:** 10.1038/s41598-018-31794-8

**Published:** 2018-09-14

**Authors:** Sheng-Yao Huang, Jung-Lung Hsu, Kun-Ju Lin, Ho-Ling Liu, Shiaw-Pying Wey, Ing-Tsung Hsiao, For the Alzheimer’s Disease Neuroimaging Initiative, Michael Weiner, Paul Aisen, Ronald Petersen, Clifford R. Jack, William Jagust, John Q. Trojanowki, Arthur W. Toga, Laurel Beckett, Robert C. Green, Andrew J. Saykin, John Morris, Leslie M. Shaw, Enchi Liu, Tom Montine, Ronald G. Thomas, Michael Donohue, Sarah Walter, Devon Gessert, Tamie Sather, Gus Jiminez, Danielle Harvey, Matthew Bernstein, Nick Fox, Paul Thompson, Norbert Schuff, Charles DeCarli, Bret Borowski, Jeff Gunter, Matt Senjem, Prashanthi Vemuri, David Jones, Kejal Kantarci, Chad Ward, Robert A. Koeppe, Norm Foster, Eric M. Reiman, Kewei Chen, Chet Mathis, Susan Landau, Nigel J. Cairns, Erin Householder, Lisa Taylor Reinwald, Virginia Lee, Magdalena Korecka, Michal Figurski, Karen Crawford, Scott Neu, Tatiana M. Foroud, Steven G. Potkin, Li Shen, Faber Kelley, Sungeun Kim, Kwangsik Nho, Zaven Kachaturian, Richard Frank, Peter J. Snyder, Susan Molchan, Jeffrey Kaye, Joseph Quinn, Betty Lind, Raina Carter, Sara Dolen, Lon S. Schneider, Sonia Pawluczyk, Mauricio Beccera, Liberty Teodoro, Bryan M. Spann, James Brewer, Helen Vanderswag, Adam Fleisher, Judith L. Heidebrink, Joanne L. Lord, Sara S. Mason, Colleen S. Albers, David Knopman, Kris Johnson, Rachelle S. Doody, Javier Villanueva Meyer, Munir Chowdhury, Susan Rountree, Mimi Dang, Yaakov Stern, Lawrence S. Honig, Karen L. Bell, Beau Ances, Maria Carroll, Sue Leon, Mark A. Mintun, Stacy Schneider, Angela Oliver, Daniel Marson, Randall Griffith, David Clark, David Geldmacher, John Brockington, Erik Roberson, Hillel Grossman, Effie Mitsis, Leyla deToledo-Morrell, Raj C. Shah, Ranjan Duara, Daniel Varon, Maria T. Greig, Peggy Roberts, Marilyn Albert, Chiadi Onyike, Daniel D’Agostino, Stephanie Kielb, James E. Galvin, Dana M. Pogorelec, Brittany Cerbone, Christina A. Michel, Henry Rusinek, Mony J. de Leon, Lidia Glodzik, Susan De Santi, P. Murali Doraiswamy, Jeffrey R. Petrella, Terence Z. Wong, Steven E. Arnold, Jason H. Karlawish, David Wolk, Charles D. Smith, Greg Jicha, Peter Hardy, Partha Sinha, Elizabeth Oates, Gary Conrad, Oscar L. Lopez, MaryAnn Oakley, Donna M. Simpson, Anton P. Porsteinsson, Bonnie S. Goldstein, Kim Martin, Kelly M. Makino, M. Saleem Ismail, Connie Brand, Ruth A. Mulnard, Gaby Thai, Catherine Mc Adams Ortiz, Kyle Womack, Dana Mathews, Mary Quiceno, Ramon Diaz Arrastia, Richard King, Myron Weiner, Kristen Martin Cook, Michael DeVous, Allan I. Levey, James J. Lah, Janet S. Cellar, Jeffrey M. Burns, Heather S. Anderson, Russell H. Swerdlow, Liana Apostolova, Kathleen Tingus, Ellen Woo, Daniel H. S. Silverman, Po H. Lu, George Bartzokis, Neill R. Graff Radford, Francine Parfitt, Tracy Kendall, Heather Johnson, Martin R. Farlow, Ann Marie Hake, Brandy R. Matthews, Scott Herring, Cynthia Hunt, Christopher H. van Dyck, Richard E. Carson, Martha G. MacAvoy, Howard Chertkow, Howard Bergman, Chris Hosein, Sandra Black, Bojana Stefanovic, Curtis Caldwell, Ging Yuek Robin Hsiung, Howard Feldman, Benita Mudge, Michele Assaly, Dick Trost, Charles Bernick, Donna Munic, Diana Kerwin, Marek Marsel Mesulam, Kristine Lipowski, Chuang Kuo Wu, Nancy Johnson, Carl Sadowsky, Walter Martinez, Teresa Villena, Raymond Scott Turner, Kathleen Johnson, Brigid Reynolds, Reisa A. Sperling, Keith A. Johnson, Gad Marshall, Meghan Frey, Jerome Yesavage, Joy L. Taylor, Barton Lane, Allyson Rosen, Jared Tinklenberg, Marwan N. Sabbagh, Christine M. Belden, Sandra A. Jacobson, Sherye A. Sirrel, Neil Kowall, Ronald Killiany, Andrew E. Budson, Alexander Norbash, Patricia Lynn Johnson, Thomas O. Obisesan, Saba Wolday, Joanne Allard, Alan Lerner, Paula Ogrocki, Leon Hudson, Evan Fletcher, Owen Carmichael, John Olichney, Smita Kittur, Michael Borrie, T. Y. Lee, Rob Bartha, Sterling Johnson, Sanjay Asthana, Cynthia M. Carlsson, Adrian Preda, Dana Nguyen, Pierre Tariot, Stephanie Reeder, Vernice Bates, Horacio Capote, Michelle Rainka, Douglas W. Scharre, Maria Kataki, Anahita Adeli, Earl A. Zimmerman, Dzintra Celmins, Alice D. Brown, Godfrey D. Pearlson, Karen Blank, Karen Anderson, Robert B. Santulli, Tamar J. Kitzmiller, Eben S. Schwartz, Kaycee M. Sink, Jeff D. Williamson, Pradeep Garg, Franklin Watkins, Brian R. Ott, Henry Querfurth, Geoffrey Tremont, Stephen Salloway, Paul Malloy, Stephen Correia, Howard J. Rosen, Bruce L. Miller, Jacobo Mintzer, Kenneth Spicer, David Bachman, Elizabether Finger, Stephen Pasternak, Irina Rachinsky, John Rogers, Andrew Kertesz, Nunzio Pomara, Raymundo Hernando, Antero Sarrael, Susan K. Schultz, Laura L. Boles Ponto, Hyungsub Shim, Karen Elizabeth Smith, Norman Relkin, Gloria Chaing, Lisa Raudin, Amanda Smith, Kristin Fargher, Balebail Ashok Raj

**Affiliations:** 1 Department of Medical Imaging and Radiological Sciences and Healthy Aging Research Center, Chang Gung University, Taoyuan, Taiwan, ROC; 2 Department of Neurology and Dementia Center, Linkou Chang Gung Memorial Hospital, Taoyuan, Taiwan, ROC; 3 Graduate Institute of Humanities in Medicine, Taipei Medical University, Taipei, Taiwan, ROC; 4 Department of Nuclear Medicine and Molecular Imaging Center, Linkou Chang Gung Memorial Hospital, Taoyuan, Taiwan, ROC; 5 Department of Imaging Physics, University of Texas MD Anderson Cancer Center, Houston, USA; 6 UC San Francisco, San Francisco, USA; 7 UC San Diego, La Jolla, USA; 8 Mayo Clinic, Rochester, MN USA; 9 UC Berkeley, Berkeley, San Francisco, USA; 10 University of Pennsylvania, Philadelphia, PA USA; 11 USC School of Medicine, Los Angeles, CA USA; 12 University of California, Davis Sacramento, Sacramento, CA USA; 13 Brigham and Women’s Hospital, Boston, MA USA; 14 Indiana University, Bloomington, IN USA; 15 University of Washington, Louis, USA; 16 Janssen Alzheimer Immunotherapy, San Francisco, USA; 17 University of Washington, Seattle, WA USA; 18 University of London, London, UK; 19 University of Michigan, Ann Arbor, MI USA; 20 University of Utah, Salt Lake City, UT USA; 21 Banner Alzheimer’s Institute, Phoenix, AZ USA; 22 University of Pittsburgh, Pittsburgh, PA USA; 23 University of Pennsylvania School of Medicine, Philadelphia, PA USA; 24 University of California Irvine, Irvine, CA USA; 25 Khachaturian, Radebaugh & Associates, Inc, Maryland, USA; 26 Ronald and Nancy Reagan’s Research Institute, Chicago, IL USA; 27 General Electric, Canada, USA; 28 Brown University, Providence, RI USA; 29 National Institute on Aging, Baltimore, Maryland USA; 30 National Institutes of Health, Sacaton, USA; 31 Oregon Health and Science University, Portland, OR USA; 32 University of Southern California, Los Angeles, CA USA; 33 Baylor College of Medicine, Houston, TX USA; 34 Columbia University Medical Center, New York, NY USA; 35 University of Alabama Birmingham, Birmingham, AL USA; 36 Mount Sinai School of Medicine, New York, NY USA; 37 Rush University Medical Center, Chicago, IL USA; 38 Wien Center, Miami Beach, FL USA; 39 Johns Hopkins University, Baltimore, MD USA; 40 New York University, New York, NY USA; 41 Duke University Medical Center, Durham, NC USA; 42 University of Kentucky, Lexington, KY USA; 43 University of Rochester Medical Center, Rochester, NY USA; 44 University of Texas Southwestern Medical School, Dallas, TX USA; 45 Emory University, Atlanta, GA USA; 46 University of Kansas, Medical Center, Kansas City, KS USA; 47 Mayo Clinic, Jacksonville, Florida USA; 48 Yale University School of Medicine, New Haven, CT USA; 49 McGill Univ., Montreal Jewish General Hospital, Montreal, QC Canada; 50 Sunnybrook Health Sciences, Toronto, ON Canada; 51 U.B.C. Clinic for AD & Related Disorders, Vancouver, BC Canada; 52 Cognitive Neurology St. Joseph’s, Ontario, ON Canada; 53 Cleveland Clinic Lou Ruvo Center for Brain Health, Las Vegas, NV USA; 54 Northwestern University, Evanston, IL USA; 55 Premiere Research Inst, West Palm Beach, FL USA; 56 Georgetown University Medical Center, Washington, DC USA; 57 Stanford University, Stanford, CA USA; 58 Banner Sun Health Research Institute, Sun City, AZ USA; 59 Boston University, Boston, MA USA; 60 Howard University, Washington, DC USA; 61 Case Western Reserve University, Cleveland, OH USA; 62 Neurological Care of CNY, Liverpool, NY USA; 63 Parkwood Hospital, London, ON Canada; 64 University of Wisconsin, Madison, WI USA; 65 Dent Neurologic Institute, Amherst, NY USA; 66 Ohio State University, Columbus, OH USA; 67 Albany Medical College, Albany, NY USA; 68 Hartford Hosp, Olin Neuropsychiatry Research Center, Hartford, CT USA; 69 Dartmouth Hitchcock Medical Center, Lebanon, NH USA; 70 Wake Forest University Health Sciences, Winston-Salem, NC USA; 71 Rhode Island Hospital, Providence, RI USA; 72 Butler Hospital, Providence, RI USA; 73 Medical University South Carolina, Charleston, SC USA; 74 St. Joseph’s Health Care, Irvine, CA USA; 75 Nathan Kline Institute, Orangeburg, NY USA; 76 University of Iowa College of Medicine, Iowa City, Iowa City, IA USA; 77 Cornell University, Ithaca, NY USA; 78 University of South Florida: USF Health Byrd Alzheimer’s Institute, Tampa, FL USA

## Abstract

The change in hypometabolism affects the regional links in the brain network. Here, to understand the underlying brain metabolic network deficits during the early stage and disease evolution of AD (Alzheimer disease), we applied correlation analysis to identify the metabolic connectivity patterns using ^18^F-FDG PET data for NC (normal control), sMCI (stable MCI), pMCI (progressive MCI) and AD, and explore the inter- and intra-hemispheric connectivity between anatomically-defined brain regions. Regions extracted from 90 anatomical structures were used to construct the matrix for measuring the inter- and intra-hemispheric connectivity. The brain connectivity patterns from the metabolic network show a decreasing trend of inter- and intra-hemispheric connections for NC, sMCI, pMCI and AD. Connection of temporal to the frontal or occipital regions is a characteristic pattern for conversion of NC to MCI, and the density of links in the parietal-occipital network is a differential pattern between sMCI and pMCI. The reduction pattern of inter and intra-hemispheric brain connectivity in the metabolic network depends on the disease stages, and is with a decreasing trend with respect to disease severity. Both frontal-occipital and parietal-occipital connectivity patterns in the metabolic network using ^18^F-FDG PET are the key feature for differentiating disease groups in AD.

## Introduction

Alzheimer’s disease (AD) is a neurodegenerative disease with characterization of deficits in progressive memory loss, cognitive and behaviour functions. Mild cognitive impairment (MCI) is a predromal stage of AD, displaying cognitive deficit but neither marked functional impairment nor satisfying established clinical criteria for dementia or probable AD^1^. However, not all MCI patients may eventually progress to AD (progressive MCI, pMCI)^2^, and some remain unchanged (stable MCI, sMCI), or are recovered from^3^. Therefore, differential diagnosis of MCI types and earlier diagnosis of AD and prediction of disease evolution are difficult^4^ but important for developing disease modifying treatment^5^.

Neurodegeneration due to an underlying physiopathology can be captured by imaging biomarkers from amyloid-specific tracers^6^, tau and the glucose metabolism from 18F-fluorodeoxyglucose (18F-FDG) in positron emission tomography (PET) for neuronal injury and dysfunction^7^. Amyloid PET imaging has provided useful information in detecting the accumulation of amyloid plaque and early neurodegeneration in the human brain^8^. Some studies have shown MCI subjects possess characteristic AD pathology including Aβ plaques and neurofibillary tangles^9^. In longitudinal amyloid imaging studies, amyloid imaging has been used to predict clinical progression to AD and the amyloid deposition rate in patients with MCI. In the past, some research groups have studied the characteristic imaging patterns of cerebral perfusion and metabolism using FDG PET in MCI and AD^10^. Progression of the disease has also been shown to be associated with a continuing decrease in glucose metabolism in affected brain regions including the parietotemporal^11^ and posterior cingulate cotices^12^.

Recent studies suggested the human brain connectome can be mapped using neuroimaging data^13–16^. Brain network analysis using neuroimaging methods based on graph theory has been applied in studying functional or structural connectivity in human brain network analysis for various neurodegenerative diseases including AD^13,17^. Among all, regional interconnectivity of glucose metabolism based on interregional correlation analysis has attracted increasing attention due to its capability in providing useful information for assessing functional neural systems^18^. Brain networks among anatomically distinct regions are functionally connected^19^ and correlation matrices of regional metabolic rates have been widely applied to infer connectivity^15^. Other methods have also been proposed to calculate the connectivity, including sparse inverse covariance estimation^20,21^ and multivariate decomposition approaches^22^, hierarchical multivariate covariance analysis^11,23,24^, and maximum likelihood estimation^25^. Moreover to improve accuracy, voxel-based multivariate statistical methods with FDR or FWE-corrections, and inclusion of the clinical factors as covariates were used in the statistical model^26–28^.

Normal human brains tend to have strong connection within lobes than between- lobes^29^, and also higher connectivity within contralateral homologues^30^. A previous study using metabolic network analysis in AD showed weaker between lobe connectivity than within-lobe, and weaker between-hemisphere connectivity as compared to normal control (NC)^20^. To understand the underlying brain network deficits during the early stage and disease evolution of AD, it is important to have a characteristic pattern of inter and intra-hemisphere metabolic connectivity for various stages of AD, including sMCI and pMCI. Specifically in this paper, we applied correlation analysis to identify the metabolic connectivity patterns using FDG-PET data for NC, sMCI, pMCI and AD, and explored the inter- and intra-hemispheric connectivity between anatomically-defined brain regions. In addition to the usual metabolic distribution patterns, a link of network connectivity to the disease evolution was also investigated.

## Results

### Three-Dimensional Views of Mean FDG Uptake

**Figure 1:**
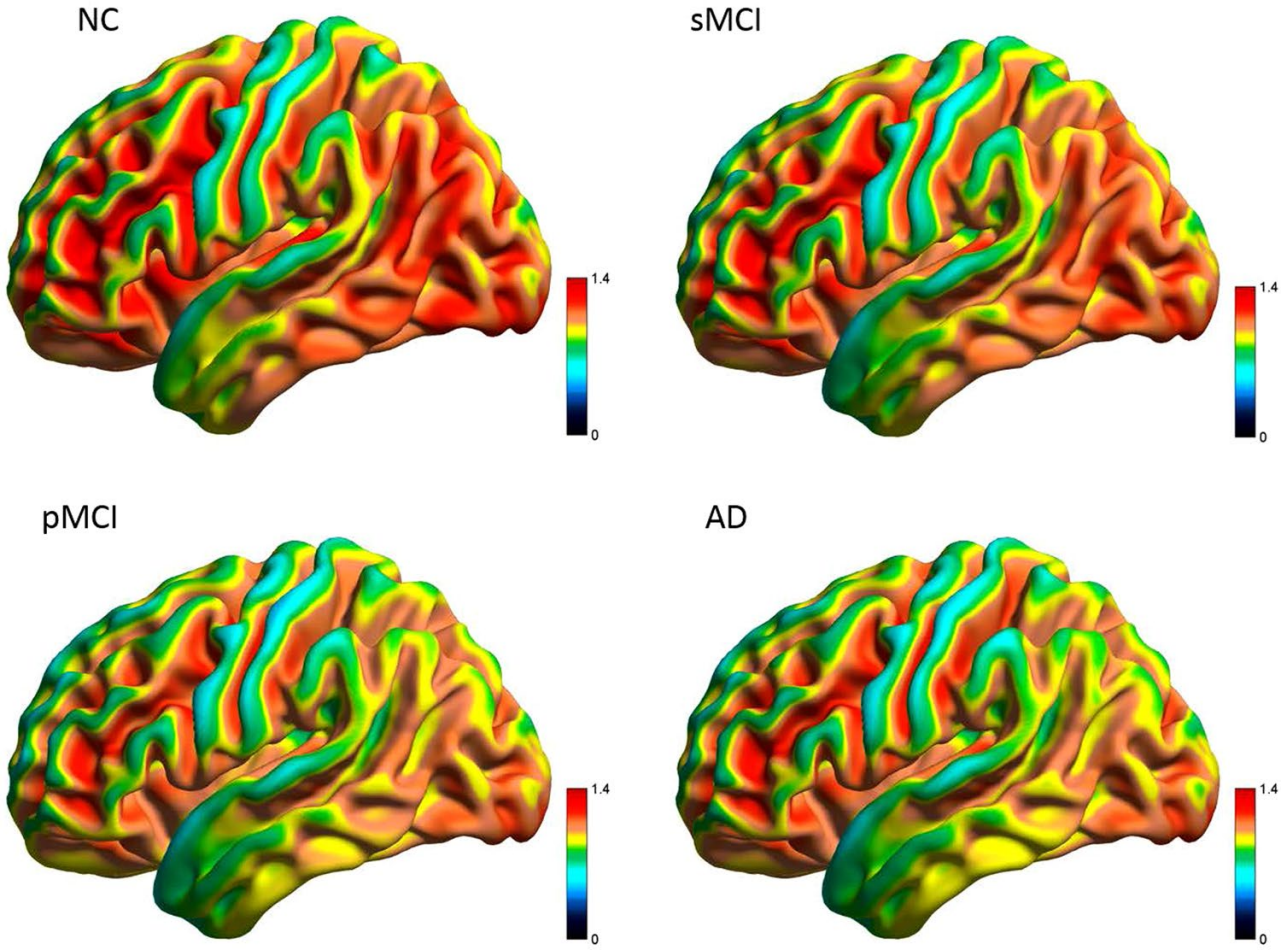
Three-dimensional visualization of mean SUVR uptake of FDG-PET in NC, sMCI, pMCI and AD from a lateral view.

The average FDG SUVR images for all groups are shown in Fig. 1. As compared to NC, an overall reduction of metabolism in the whole brain was seen in AD, especially in frontal, parietal, temporal and occipital regions. A similar distribution pattern to that in AD was observed in pMCI, while the regional metabolic pattern in sMCI was overall similar to but relatively lower than that in NC, and in particular, within the parietal and temporal cortices.

### Group Comparison of Significant differences

**Figure 2:**
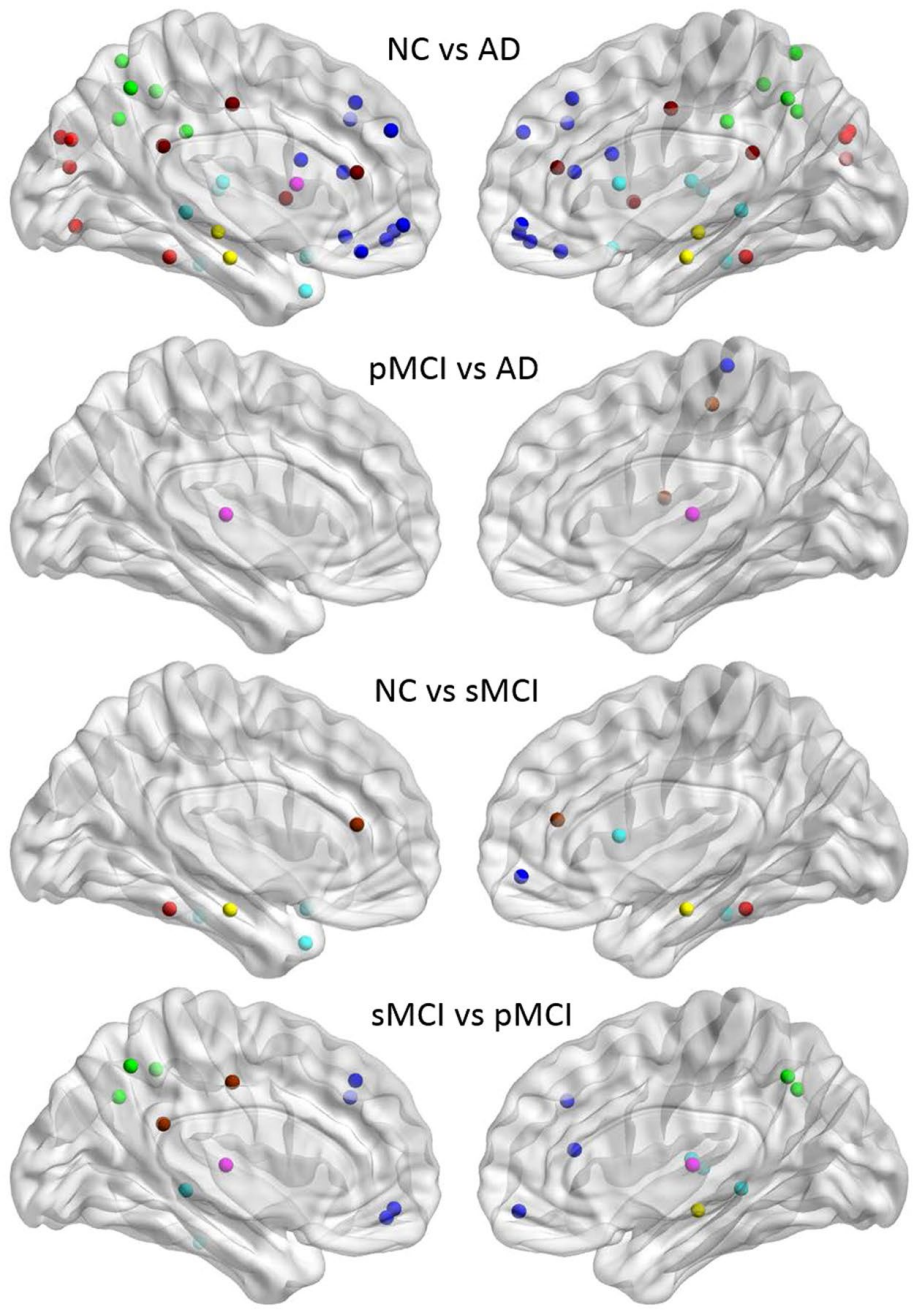
Nodes with significant SUVR difference in group comparison of NC, pMCI, AD and sMCI. Abbreviations for the regions are described in Supplementary Table 2. Regional color representations are as follows: deep blue for frontal; light blue for temporal; green, for parietal; red for occipital; pink for thalamus, pallidum, caudate, putamen, amygdala; yellow for hippocampus; deep red for other regions.

The regional SUVR comparison between two groups was calculated by a two-sample t-test (p  <  0.01) for all VOIs. Figure 2 displays the VOIs with significant differences between two groups for NC vs. AD, pMCI vs. AD, NC vs. sMCI and sMCI vs. pMCI. As expected, more significant hypometabolic regions were observed in AD as compared to NC in the whole brain, and in particular, in the regions of the frontal, temporal and parietal lobes from both hemispheres. There are only few VOIs with significant SUVR difference between pMCI and AD including bilateral thalamus, right rolandic operculum, right postcentral gyrus, and right paracentral lobule. As compared to NC, sMCI was hypometabolic only in the cingulum, parahippocampal, fusiform, and temporal lobe regions. The regions with significantly different metabolism between sMCI and pMCI were located in the frontal, cingulum, temporal, thalamus, parietal, angular, and precuneus.

### Inter-hemispheric correlation coefficients matrices

**Figure 3:**
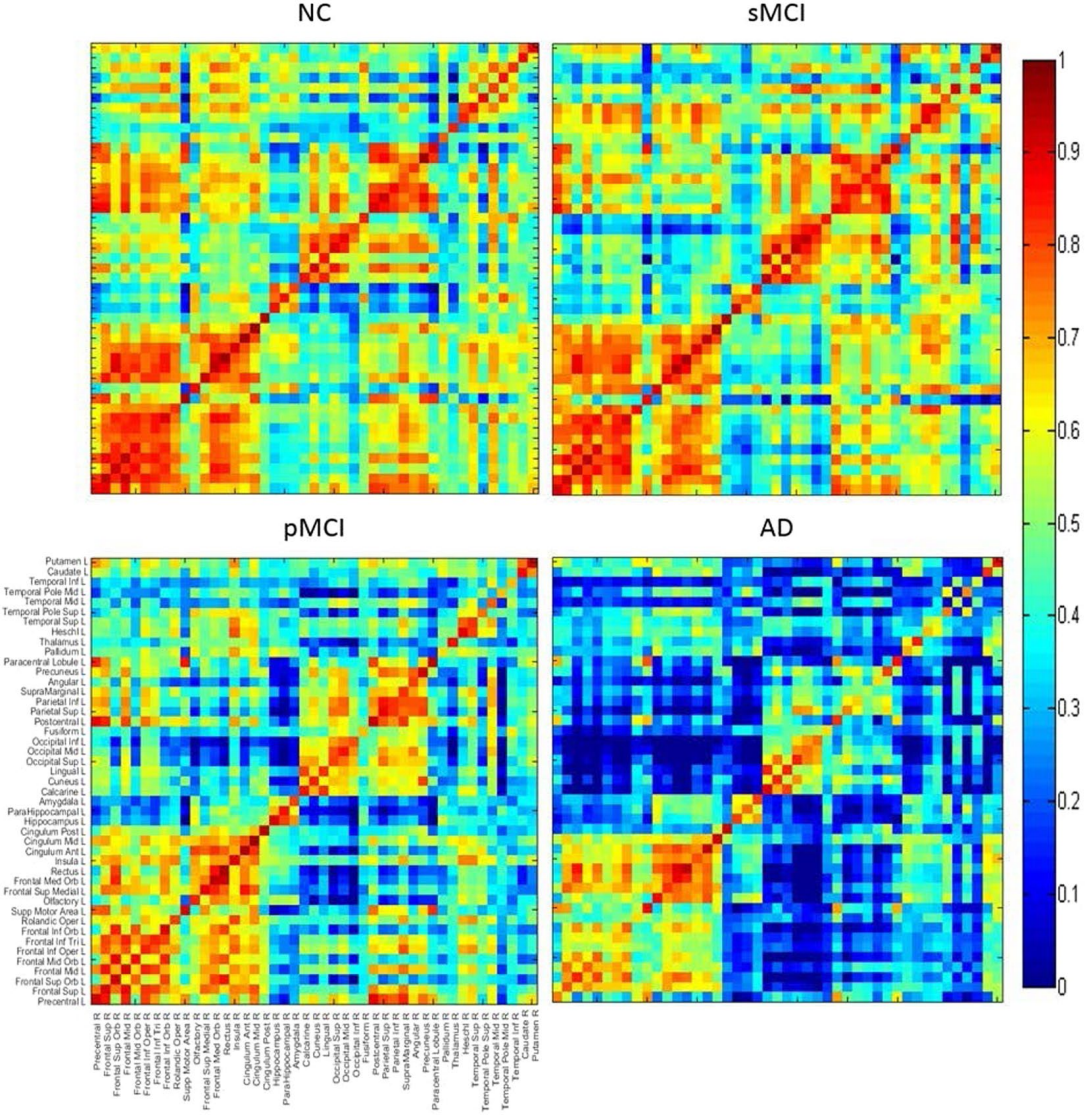
Matrices of correlation coefficients between the right and left hemisphere (ordinate) brain regions. The inter-hemispheric connectivity is illustrated from the matrices of correlation coefficients between the right hemisphere (abscissa) and left hemisphere (ordinate) brain regions for NC, sMCI, pMCI and AD.

Figure 3 displays the correlation coefficients matrices between the left (ordinate) and right (abscissa) hemispheres. Among the four groups, the main difference in the connection between hemispheres were in the temporal lobe, the parietal lobe, putamen, caudate, thalamus, and the occipital lobe. Overall, more connections in NC were observed as compared to AD, and in particular, AD had obviously decreased connections between the frontal lobe and other regions. The correlation between the right occipital and the left temporal regions was slightly higher in sMCI than in NC. Interestingly, the correlation within the frontal lobes was relatively increased in AD as compared to pMCI.

### Brain connectivity graph

**Figure 4:**
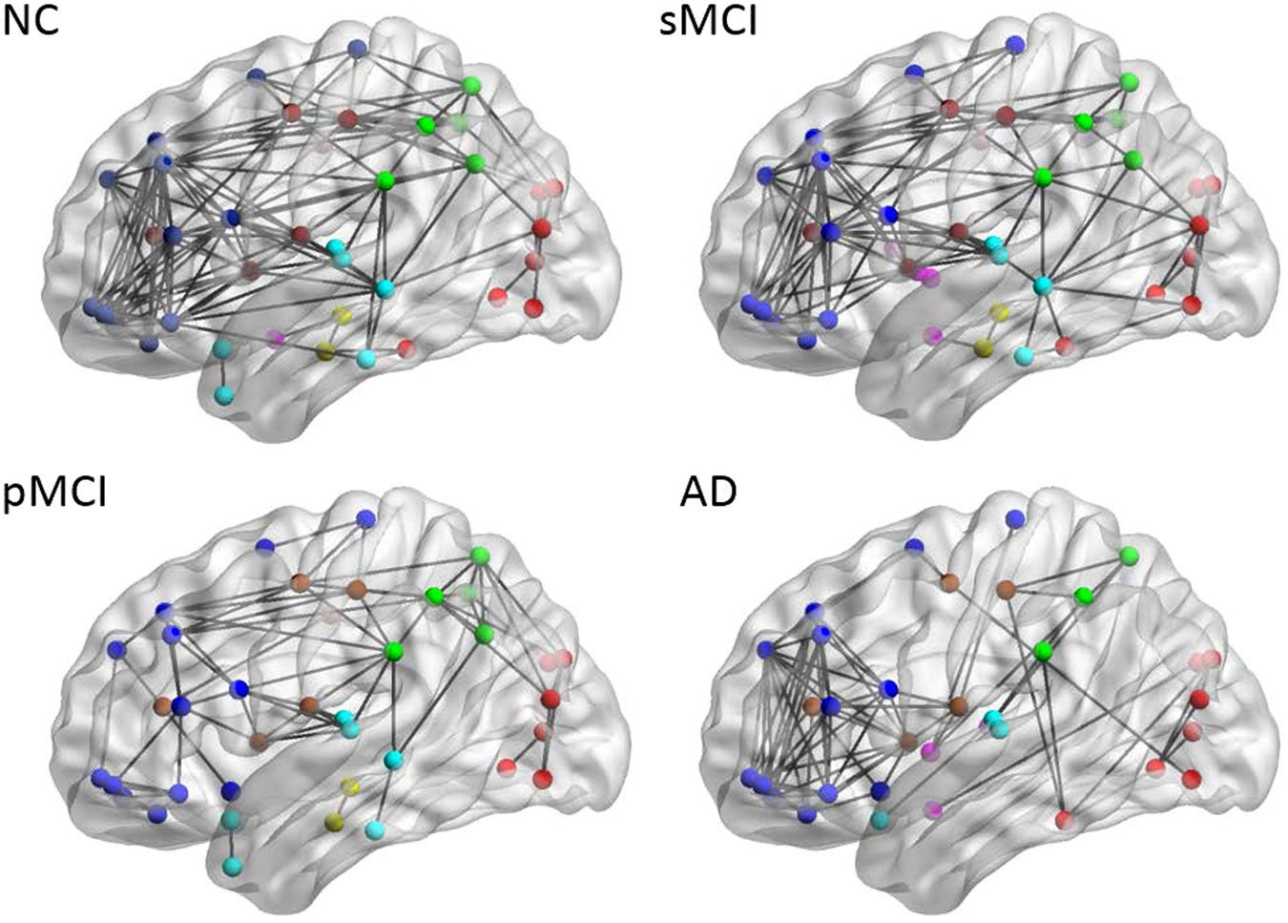
Brain connectivity graphs in NC, sMCI, pMCI and AD from a lateral view. Brain connectivity graphs were visualized in 3D view for four groups and obtained using a correlation coefficient threshold for each group. The intra-hemispheric connections were indicated by black lines and nodes in each region by the color dots.

Figure 4 illustrates the connectivity graph for each group within the same hemisphere from the binary matrices obtained by a predetermined threshold. To reduce the display complexity in the intra-hemispheric network, the lowest threshold value 0.78 for the connectivity map in NC containing 90 nodes was selected for all groups^31^. The resulting number of nodes in the connectivity network for each group is 90, 88, 85 and 86, while the resulting number of edges is 138, 114, 73, and 78, for NC, sMCI, pMCI and AD, respectively. A significant reduction of intra-hemispheric connections was observed in pMCI and AD. As compared to NC, the number of edges in AD decreased mainly in regions including the frontal lobe, temporal lobe, parietal lobe, occipital lobe, and central region of precentral gyrus, supplementary motor area, and thalamus.

### Inter-hemispheric connectivity network

**Figure 5:**
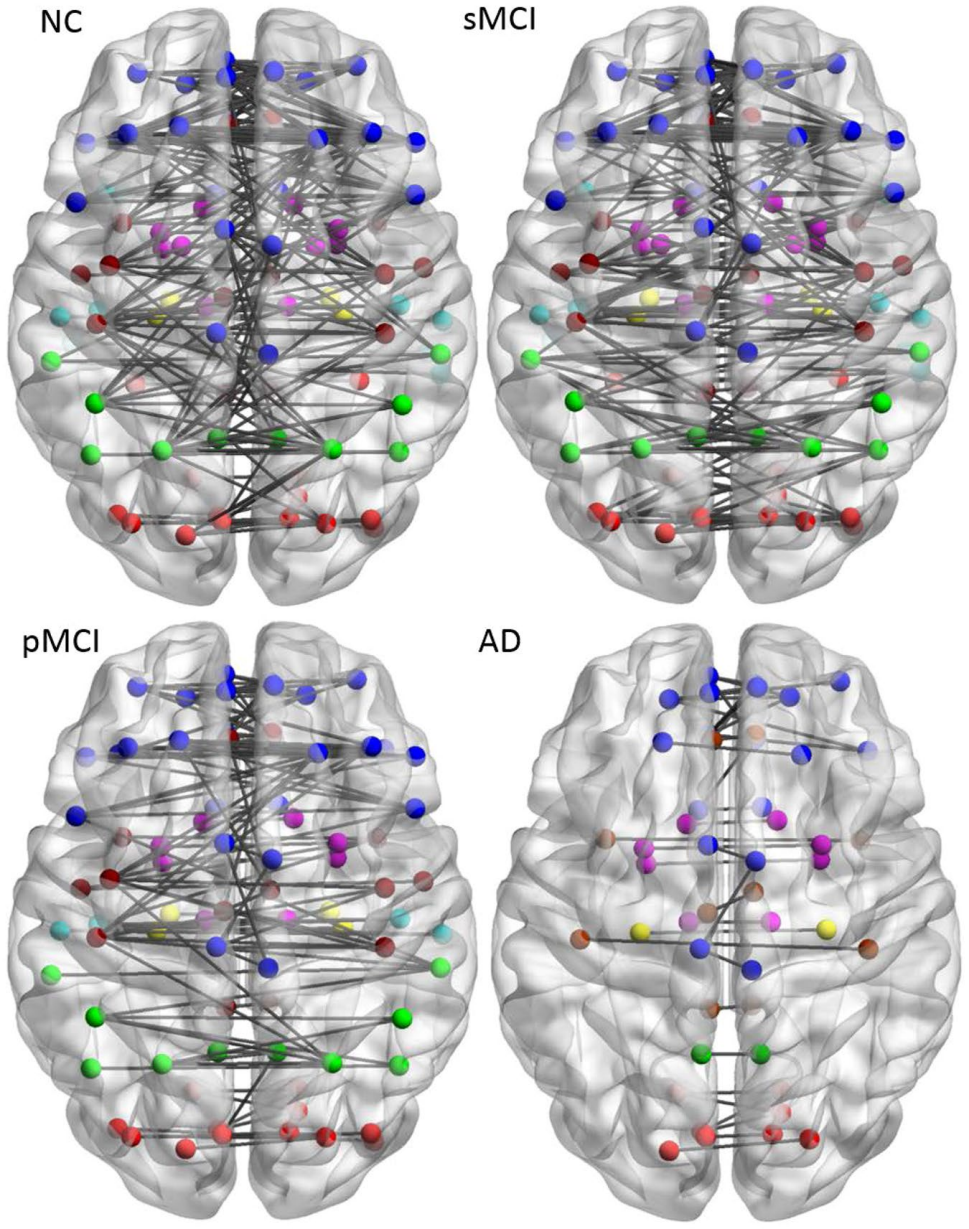
Brain connectivity graphs in NC, pMCI, AD and sMCI. Brain inter- hemispheric connectivity graphs were visualized for four groups and obtained from thresholding the correlation coefficient matrix for each group. The inter-hemispheric connections were indicated by black lines and nodes by the color dots (deep blue for frontal; light blue for temporal; green for parietal; red for occipital; pink for thalamus, pallidum, caudate, putamen, amygdala; yellow for hippocampus; deep red for other regions).

Figure 5 illustrates the axial view of the inter-hemispheric connectivity network built from a binary matrix as measured from the same correlation coefficient threshold (0.78) for all four groups. The number of inter-hemispheric edges in NC and sMCI is similar (180 and 169, respectively), but it dropped significantly to 105 and 36 for pMCI and AD. The patterns of inter-hemispheric connectivity were similar in NC and sMCI but later showed reduced inter-lobe connections and more links in the temporal lobe, including fusiform, mid-temporal, and inferior temporal. The network connectivity for sMCI was significantly higher than that for pMCI in the parietal and parietal-occipital lobes while it showed similar patterns to NC in the parietal region.

## Discussion

Using VOI-based connectivity analysis from ^18^FDG PET images, we investigated the characteristic patterns of the inter- and intra-hemispheric metabolic network among the groups of NC, stable and progressive MCI, and AD. The results of the correlation matrix indicated the regional SUVR correlations decreased between four main regions for different disease stages: the frontal lobe, occipital lobe, parietal lobe, and the temporal lobe. As in previous studies, our result found the major hypometabolic difference among the groups is located in the parietal and temporal regions^32^, and these regions were reported to predict clinical progression of normal elderly into MCI^33^. Our results also showed connectivity patterns from the metabolic network display the decreasing inter- and intra-hemispheric trends for the disease stages of NC, sMCI, pMCI and AD.

Previous studies reported AD as a disconnection syndrome of inter and intra-hemispheric coherences with functional disruption in the brain^17,34^. Our results (see Supplementary Table S3) also indicated network changes with decreased connections and linking patterns that depends on the disease stage. In addition, as shown in Supplementary Table S4, as compared to HC, the number of edges in sMCI increased 1.5 times in the temporal-occipital network, but reduced 0.5 times in the parietal-occipital network. This phenomenon could be due to the compensatory mechanisms as mentioned before^35^.

In our study, there are differences in network connection between NC and MCI mainly located in the fusiform, middle temporal, and inferior temporal regions. Previous studies reported similar finding in network changes in these regions^29^. People with MCI have a high risk of developing AD. However, it is not clear how the metabolic connectivity pattern differs between sMCI and pMCI among MCI patients. A previous study just indicated the reduction in glucose metabolism in the parietal lobe for MCI^29^. From our result (Fig. 4), a specific network pattern with connection from parietal to occipital regions was found only for pMCI but not for sMCI. Moreover, there are 1.5 times more links in the parietal-occipital network for pMCI as compared to sMCI. These patterns could be possible due to compensatory effect.

For comparison, we also conducted a voxel-wise network analysis with multiple regression (FWE correction p  <  0.05, and inclusion of the MMSE and gender as covariates) as in Nobili *et al*.^26^ and Carbonell *et al*.^28^ for both sMCI and pMCI. The preliminary result has been included as supplementary data (Figs S1 and S2). From the voxel-wise analysis, as compared to the ROI-based network analysis, and shown in Figs S1 and S2 of the supplementary data, both methods display different connectivity patterns from occipital to temporal and to parietal between sMCI and pMCI.

After comparing the network links between the 90 nodes within the brain, the lost intra-hemispheric connections observed in patients as compared to NCs can be divided into two key patterns. First, functional connectivity between the parietal lobes^11^ (superolateral and precuneus) and the occipital lobes was only found in NC and pMCI, but not in sMCI (Fig. 4). Second, more connectivity between the temporal lobes and occipital lobes was observed in sMCI as compared to NC (Fig. 4). The pattern of connectivity changes in the temporal lobe (from temporal-frontal to temporal-occipital) for AD is well-documented^36^ and similar pattern was also observed in a previous study on a seed-based metabolic correlation analysis^29^, and hierarchical multivariate covariance analysis for patients with low and high beta-amyloid burdens^11,24^, where they found metabolic connectivity change in the temporal-parietal regions usually exist in patients with high amyloid deposition. Similar pattern of decreased connectivity in the regions of left occipital and parietal for AD with CDR of 0.5 by using interregional correlation analysis and permutation test^16^. These two key patterns can be potentially used as biomarkers in identifying individuals of MCI at highest risk of progression to AD.

We have applied SUVR for the construction of metabolic brain network. Unlike SUV, SUVR is a relative value by normalizing the mean SUV in a target region to that in a reference region which is stable and unaffected by the process under investigation. In addition, SUVR is usually applied in longitudinal and intersubject studies^37^, and thus is suitable for metabolic network application.

Despite the large number of PET images in NC and AD, the design of the present study is not without limitations. First, an optimal and effective selection of the threshold for correlation value used in the correlation matrix is still a challenging issue. This study, we assumed the connectivity network should have all connections (90 nodes) in NC and we select the highest correlation threshold of 0.78 to show the results and to avoid inclusion of many false connections^38^. Other studies used a range of sparsity degrees from 0.5 to 0.9 as thresholds^39,40^, but this led to variable results^41^. Small-world indices were also applied to show a connectivity network^33,41^ but still no optimal solution for selecting a standard threshold. Some other limitations are associated with the connectivity analysis based on evaluation of interregional correlations^11^. To alleviate these limitations, hierarchical multivariate covariance analysis^11^ or voxel-based multivariate statistical methods with FDR or FWE-corrections, and inclusion of the clinical factors as covariates could be used in the statistical mode^26–28,42^. Therefore, future work should include thorough comparison of connectivity analysis using different approaches^11,23,26,27^, developing an individual and effective metabolic network and voxel-wise network for clinical diagnosis applications.

## Conclusion

This paper studied the patterns of inter and intra-hemisphere functional metabolic connectivity in NC, sMCI, pMCI and AD based on PET FDG data. Two major key metabolic network patterns were observed among these four groups in this study. Connection of temporal lobes to frontal or occipital is a characteristic pattern for conversion of NC to MCI, and the density of links in the parietal-occipital network is a differential biomarker from sMCI to pMCI.

## Methods

### Subjects

Data used in the preparation of this article were obtained from the Alzheimer’s Disease Neuroimaging Initiative (ADNI) database (adni.loni.usc.edu). Te ADNI was launched in 2003 as a public-private partnership with a primary goal to test whether serial magnetic resonance imaging (MRI), positron emission tomography (PET), other biological markers, and clinical and neuropsychological assessment can be combined to measure the progression of mild cognitive impairment (MCI) and early Alzheimer’s disease (AD). ADNI (ADNI ClinicalTrials.gov identifer: NCT00106899) is the result of efforts of many coinvestigators from a broad range of academic institutions and private corporations, with subjects recruited from over 50 sites across the United States and Canada. Details of the ADNI-1 and ADNI-2 protocol, timelines, study procedures and biomarkers can be found in the ADNI-1 and ADNI-2 procedures manual [https://www.adni-info.org/]. For up-to-date information, see www.adni-info.org

There were PET scans from 100 MCI subjects, 100 AD subjects, and 100 NC subjects included in this study. The 100 MCI subjects were further divided into two groups: (i) sMCI (stable MCI), if the diagnosis was MCI at all available time points, and at least for 36 months (n  =  45); (ii) pMCI (progressive MCI), if the diagnosis was MCI at baseline but progressed to AD was reported within 12 months after baseline, and without reversion to MCI or NC at any available follow-up (n  =  55)^43^. The main demographic and clinical data for each group are summarized in Supplementary Table S1. All subjects underwent thorough clinical and cognitive assessments at the time of each of their PET scans. Each subject’s cognitive evaluation included the following: (i) the MMSE to provide a global measure of mental status^44^; (ii) the Global CDR to determine the stage severity of dementia^36^. More details about all the tests can be found on the ADNI website at www.loni.ucla.edu/ADNI.

The study protocols were approved by the institutional review board of Chang Gung Memorial Hospital and ADNI (a complete list of ADNI sites is available at https://www.adni-info.org/.) and written informed consent was obtained from all participants or authorized representatives for the original data acquisition in ADNI. All the analytical methods were performed on the de-identified ADNI data. In addition, these methods were carried out in accordance with the approved guidelines.

### Image analysis

We downloaded the preprocessed FDG-PET scans from the public ADNI database (www.loni.ucla.edu/ADNI). The PET image acquisition and preprocessing protocols prior to download can be found elsewhere^2,45^. All downloaded PET data were then further processed using PMOD image analysis software (version 3.3; PMOD Technologies Ltd, Zurich, Switzerland) and spatially normalized into the Automated Anatomical Labeling (AAL) space. All images were automatically segmented into 90 anatomical structures (volumes of interest, VOIs) using the AAL atlas^45^. For the standard quantification procedure of the FDG image, the regional radioactivity concentration was first converted to standardized uptake values (SUVs)^46^. Then, the regional SUV ratio (SUVR) of the mean SUV between the target and reference regions was calculated with the entire cerebellum as the reference region^47^. Finally, each subject’s regional SUVR for each AAL structure was extracted to construct the SUVR data matrix. For each group (NC, sMCI, pMCI and AD), the data matrix had a size of M × N, where ‘M’ represents the number of subjects within each group, and ‘N’ the number of AAL structures.

### Brain network

From the network theory, a network (or graph) is a mathematical model representing a collection of nodes (or vertices) and edges (or connections) between pairs of node^48^. When considering brain networks, the network nodes should ideally represent meaningful brain regions. However, it is more common to convert the connectivity matrix to a binary matrix by retaining only the links above a certain threshold. This leads to a binary network model, where the links above the threshold are represented by 1 (presence of edge) and those below it are represented by 0 (absence of edge). In our study, a connection in a brain network is defined in terms of statistical associations between each pair of brain regions among the 90 anatomical structures^49^. The statistical association was obtained by synchronized co-variations and measured by computing their Pearson’s correlation coefficient, across subjects. Hence, an interregional Pearson’s correlation coefficient matrix (N × N, where N is the number of brain regions; here, N = 90) for the statistical connections was calculated using all pairs of anatomical structures. To obtain a binary connectivity network, a threshold is needed^13,14^. Here, various thresholds ranging from 0.5 to 0.9, in steps of 0.02, yielding a set of 21 values^39^ were applied, and that yielded a set of 21 binary connectivity matrices for each group. We further used the BrainNet Viewer (www.nitrc.org/projects/bnv/) toolbox to display connections forming the subnetwork in four groups.

### Statistical analyses

Statistical analyses were performed with the SPSS 17.0 statistical package (SPSS Statistics for Windows, version 17.0, 2008), and p values  <  0.01 were considered significant. Two-sample t-tests were used to examine the differences in clinical characteristics (age, education, weight, MMSE and CDR) scores. The regional SUVRs among the four groups (NC, sMCI, pMCI, AD) were also compared using the multiple comparison test.

All methods were performed in accordance with the relevant ethical guidelines and regulations as stated in the first section of Methods.

## Electronic supplementary material

Supplementary Information

## Data Availability

PET images were downloaded online from ADNI (https://ida.loni.usc.edu) and further processed locally (see Image Analysis above). Processed ADNI data are not publicly available for download but are available from the corresponding author.
