# Supplementary material for: Characteristic patterns of inter- and intra-hemispheric metabolic connectivity in patients with stable and progressive mild cognitive impairment and Alzheimer’s disease
Source: Sci Rep. 2018 Sep 14;8:13807. doi: 10.1038/s41598-018-31794-8 (PMC6138637; doi:10.1038/s41598-018-31794-8)
Supplement: Supplementary file 1 — Supplementary Information [file 41598_2018_31794_MOESM1_ESM.pdf]

Characteristic patterns of inter- and intra-hemispheric metabolic connectivity in patients with stable and progressive mild cognitive impairment and Alzheimer's disease

Sheng-Yao Huang[1] , Jung-Lung Hsu[2,3], Kun-Ju Lin[1,4], Ho-Ling Liu[5], Shiaw-Pying Wey[1] , Ing-Tsung Hsiao[1,4*] & Alzheimer's Disease Neuroimaging Initiative†

Supplementary information

Table S1. Summary of subject information. Group demographics and clinical data at 6 months follow-up.

Variable	Mean (±SD)			
	NC	pMCI	AD	sMCI
No. subjects	100	56	100	45
Gender (male/female)	61/39	35/21	58/42	32/13
Age (years)	75.6 (±4.3)	75.4 (±6.4)	76 (±6.7)	77.1 (±7)
Education (years)	15.1 (±3.2)	15.8 (±2.9)	15.5 (±3.1)	14.8 (±3.3)
Weight (kg)	75.5 (±13.6)	72.5 (±15.9)	76.1 (±15.4)	79.1 (±13)
MMSE	29.1 (±0.9)	26 (±2.7)	21.9 (±4)	27 (±4.6)
Global CDR	0.01 (±0.1)	0.51 (±0.1)	0.93 (±0.5)	0.47 (±0.1)

Table S2. Nodes with significantly different SUVR in group comparison of NC, pMCI, AD and sMCI.

Comparsion	Features (90 nodes)	No.
NC-sMCI	16, 20, 28, 40, 42, 43, 58, 61, 65, 73, 87, 88	12
NC-pMCI	1-28, 30-34, 36, 38-43, 47-54, 56-66, 68, 70, 71, 73, 75-79, 81-86, 88-89	76
NC-AD	2-8, 12-20, 23, 25-28, 30-34, 38-44, 47-52, 57-65, 68, 70, 71, 73, 75-79, 83-88	63
sMCI-pMCI	2-5, 17-18,31, 33, 34, 37, 41, 43, 49, 50, 52, 64, 78-79, 82-84	21
sMCI-AD	4, 18, 20, 30, 31, 33, 34, 41, 43, 63, 64, 76, 78, 79, 86, 88	16
pMCI-AD	37, 54, 74, 80, 82	5

Table S3. Number of edges for inter and intra-hemispheric network for each group.

Disease (No. Nodes)	No. Edges			
	L-L	R-R	L-R	Total
NC (90)	138	111	180	429
sMCI (88)	114	107	169	390
pMCI (85)	73	70	105	248
AD (86)	78	57	36	171

L-L: Left intra-hemisphere, **R-R:** Right intra-hemisphere, and **L-R: Left and right i**nter-hemisphere

Table S4. Number of edges rate for Left Hemispheric network.

Left Hemispheric (No.edges rate)	(sMCI-NC)/NC	(pMCI-sMCI)/sMCI
temportal-occipital	1.5	-1
temportal-frontial	-0.6	-0.5
parietal-occipital	-0.5	1.5

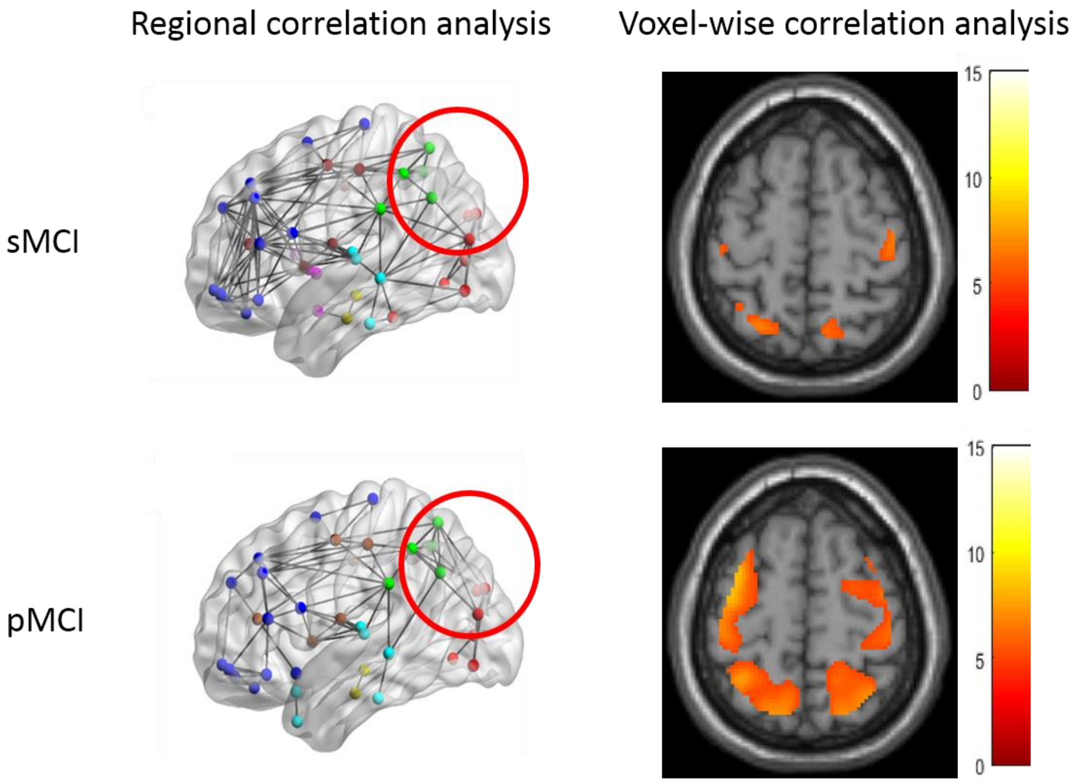

Figure S1. Comparison between between regional and voxel-wise correlation analysis in parietal lobe of sMCI and pMCI. The right side was showed our finding result. Another side, we used voxel-wise correlation analsis and the significance threshold: $p<0.05$, correlation for multiple comparison (FWE with MMSE). The chose region (red cicle part) is left occipital-parietal connectivity.

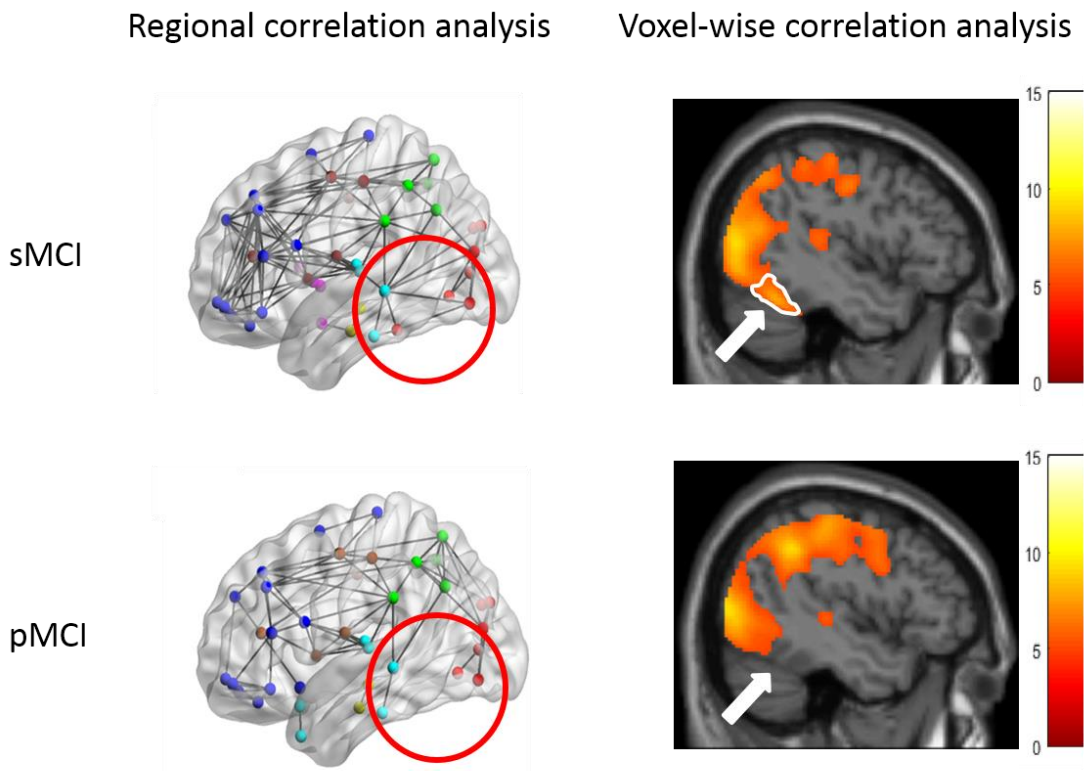

Figure S2. Comparison between between regional and voxel-wise correlation analysis in temporal lobe of sMCI and pMCI. The right side was showed our finding result. Another side, we used voxel-wise correlation analsis and the significance threshold: p<0.05, correlation for multiple comparison (FWE with MMSE). The chose region (red cicle part) is left occipital-temporal connectivity. The white areas is temporal lobe.
